# On-Reading (Chinese-Style Pronunciation) Predominance Over Kun-Reading (Native Japanese Pronunciation) in Japanese Semantic Dementia

**DOI:** 10.3389/fnhum.2021.700181

**Published:** 2021-08-05

**Authors:** Yasuhisa Sakurai, Yumiko Uchiyama, Akitoshi Takeda, Yasuo Terao

**Affiliations:** ^1^Department of Neurology, Mitsui Memorial Hospital, Tokyo, Japan; ^2^Department of Neurology, Tokyo Women’s Medical University, Tokyo, Japan; ^3^Department of Neurology, Kudanzaka Hospital, Tokyo, Japan; ^4^Department of Neurology, Osaka City University Graduate School of Medicine, Osaka, Japan; ^5^Department of Cell Physiology, Faculty of Medicine, Kyorin University, Tokyo, Japan

**Keywords:** semantic dementia, Gogi aphasia, surface dyslexia, Kanji, Kana, On-reading

## Abstract

Japanese kanji (morphograms) have two ways of reading: *on*-reading (Chinese-style pronunciation) and *kun*-reading (native Japanese pronunciation). It is known that some Japanese patients with semantic dementia read kanji with *on*-reading but not with *kun*-reading. To characterize further reading impairments of patients with semantic dementia, we analyzed data from a total of 9 patients who underwent reading and writing tests of kanji and kana (Japanese phonetic writing) and *on*-*kun* reading tests containing two-character kanji words with *on*-*on* reading, *kun*-*kun* reading, and specific (so-called Jukujikun or irregular *kun*) reading. The results showed that *on*-reading preceding (pronouncing first with *on*-reading) and *kun*-reading deletion (inability to recall *kun*-reading) were observed in nearly all patients. In the *on*-*kun* reading test, *on*-reading (57.6% correct), *kun*-reading (46.6% correct), and specific-reading (30.0% correct) were more preserved in this decreasing order (phonology-to-semantics gradient), although *on*-reading and *kun*-reading did not significantly differ in performance, according to a more rigorous analysis after adjusting for word frequency (and familiarity). Furthermore, *on*-substitution (changing to *on*-reading) errors in *kun*-reading words (27.0%) were more frequent than *kun*-substitution (changing to *kun*-reading) errors in *on*-reading words (4.0%). These results suggest that *kun*-reading is more predominantly disturbed than *on*-reading, probably because *kun*-reading and specific-reading are closely associated with the meaning of words.

## Introduction

Semantic dementia is a phenotype of frontotemporal lobar degeneration (Neary et al., 1998). The cardinal feature is semantic memory loss. Patients with semantic dementia show a severe naming and word comprehension impairment, surface dyslexia, and orthographic agraphia (inability to read or write orthographically irregular words). Characteristically, reading errors comprise regularization errors, e.g., “pint” [paint] is pronounced as [pint], like hint, and writing errors produce a homophone or pseudohomophone effect, a kind of regularization in writing, e.g., “caught” is written as “cort.”

Because Japanese kanji (Japanese morphograms) characters can be read in two ways, *on*-reading (one type of reading derived from the Chinese pronunciation) and *kun*-reading (another type of reading derived from the native Japanese language), Japanese patients with semantic dementia show *on*-*kun* confusion in reading kanji (Imura, 1943). For example, 真白 [masshiro] (*kun*-*kun* reading, meaning “pure white”) is read as [shinpaku] (*on*-*on* reading). This kind of *on*-*kun* confusion is comparable to regularization errors in Western surface dyslexia (Sakurai et al., 2006). We reported that *on*-preceding (pronouncing first with *on*-reading, irrespective of its preferred reading) and *kun*-deletion (inability to recall and recognize *kun*-reading) are characteristic of progressive Gogi (word-meaning) aphasia (Sakurai et al., 2006), a Japanese linguistic manifestation of semantic dementia (Imura, 1943).

Since this phenomenon has been observed in only two patients, we retrospectively collected additional data from another seven patients and examined whether *on*-preceding and *kun*-deletion were generally observed. Also, we analyzed the results of a special reading test given to the seven patients, consisting of two-character kanji words with *on*-*on* reading, *kun*-*kun* reading, and specific-reading or irregular word reading (so-called Jukujikun in Japanese). This test was designed to differentiate between *on*-reading predominance and *kun*-reading predominance in kanji word reading. What is the clinical significance of differentiating *on*-reading from *kun*-reading?

Japanese pupils learn how to pronounce a kanji character by *on*-reading and *kun*-reading and learn the meaning by *kun*-reading. Although *on*-reading conveys not only its phonetic value but also meaning, semantic attributes of the *on*-reading are indirectly acquired through the learning of *kun*-reading of the character and compound *on*-reading kanji words. In this sense, *on*-reading primarily conveys its phonetic value.

On the other hand, *kun*-reading is a Japanese way of reading (a kind of translation) of Chinese characters (Kindaichi, 1988). Thus, *kun*-reading impairment directly represents a loss of semantics. Moreover, given that specific-reading (Jukujikun) is the semantic assignment of Chinese characters to native Japanese words (Kindaichi, 1988) (e.g., 時雨, the combination of Chinese characters 時 meaning time and 雨 meaning rain is read as Jukujikun しぐれ [shigure], whose native Japanese meaning is occasional rain), Jukujikun can be regarded as irregular *kun*-reading (Kess and Miyamoto, 1999) whose pronunciation cannot be inferred from any reading of each component character. Therefore, in the case of Jukujikun, whole-word orthography is important in accessing pronunciation and semantics. If whole-word reading is impossible, patients should guess the pronunciation/meaning from the vague semantic context (Kess and Miyamoto, 1999) of the two component characters. In this sense, reading of Jukujikun requires direct or frequent access to semantics, and thus is more closely associated with the meaning.

On the basis of these considerations, we hypothesized that the patients read *kun-kun* reading words less accurately than *on-on* reading words, and specific-reading words less accurately than *kun-kun* reading words because the relationship between irregularity of reading and semantics is more concerned with reading specific-reading words, *kun-kun* reading words, and *on-on* reading words in this decreasing order of priority. We further investigated the neural substrate of Japanese semantic dementia, using MRI and single-photon emission computed tomography (SPECT).

## Materials and Methods

We retrospectively analyzed data on nine patients with semantic dementia, two of whom were previously reported (patients 6 and 7 in Table 1) (Sakurai et al., 2006). The data included results of reading and writing tests of single-character kanji and kana (Japanese phonetic writing) transcription (Sakurai et al., 1994). We also analyzed the data on seven patients (patients 1 to 5, 8, and 9 in Table 1) undergoing a special reading test of two-character kanji (described below). All authors were qualified neurologists and trained neuropsychologists. They first examined their patients and made a diagnosis of semantic dementia. All patients fulfilled the research diagnostic criteria for semantic dementia or the semantic variant of primary progressive aphasia (Gorno-Tempini et al., 2011) or semantic aphasia and associative agnosia (Neary et al., 1998).

**TABLE 1:** Patient profiles and neuropsychological test scores.

Patient	1	2	3	4	5	6	7	8	9
Age and sex	71M	65W	61M	60M	67M	55M	51M	67M	51M
Years of education	12	12	16	12	20	16	16	16	12
Temporal lobe atrophy	L	L	L = R	L = R	R > L	L > R	L	L = R	L > R
Time from onset^a^	2 m	4 yr	3 yr	10 yr	3 yr	9 m	4 yr	9 yr	5 yr
MMSE	22.4	n.d.	25	21	30	n.d.	22.5	27	11
**WAB**									
Content/fluency	7/8	10/8	9/9	8/9	9/9	9/9	9/8	8/8	5/8
Comprehension (/10)	9.4	8.45	9.2	7.25	9	9.35	9.35	9.25	6.3
Repetition (/10)	7.4	8.4	9.9	8.9	9.5	10	9.8	9.6	7.9
Naming total (/10)	4.5	3	3	3.2	7.7	4.8	1.2	2.5	0.4
Object naming (/60)	28	12	13	21	53	26	4	22	3
Reading total (/10)	8	7.2	6.15	6.4	9	8.1	6.15	3.4	8
Writing (/10)	9.5	6.9	8.8	7.35	9.75	9.7	9.2	8.55	6
Kanji *(/6)*	5.5	2	2	2	4.5	3.5	0.5	3	0
Kana (/6)	6	6	6	6	6	6	6	6	0
Praxis (/10)	10	9.8	8.7	8.5	9	9.7	8.4	8.33	7.3
Drawing (/30)	21	27.5	29	25.5	23	30	29	20	30
Raven’s CPM (/37)	24	34	36	28	24	31	36	36	36
**100 single-character Kanji and Kana transcription test**									
Kanji reading	100	91	94	98	100	95	73	n.d.	n.d.
Kana reading	100	100	100	100	100	100	100	n.d.	n.d.
Kanji writing	10	40	56	71	85	54	32	n.d.	n.d.
Kana writing	61	99	99	98	100	100	100	n.d.	n.d.
***On*-*Kun* reading test**									
*On*-reading (/100)	71	46	68	87	88	n.d.	n.d.	20	23
*Kun*-reading (/100)	71	29	45	63	88	n.d.	n.d.	9	21
Specific-reading (/60)	21	11	18	23	43	n.d.	n.d.	3	6

*L, left only; L = R, equally atrophied on both left and right; L > R, left side is more atrophied than right side; M, man; m, month; MMSE, Mini-mental state examination; n.d., not done; Raven’s CPM, Raven’s Colored Progressive Matrices; W, woman; WAB, Western Aphasia Battery; yr, year. ^*a*^Time of evaluation after disease onset.*

### Neuropsychological Assessment

Patients’ language function was rated with Western Aphasia Battery (Japanese edition) (Table 1). Reading and writing were assessed with 100 word single-character kanji and kana transcription (Sakurai et al., 1994), all of which are taught in the first 3 years of primary school in Japan (Supplementary Appendix 1). All kanji characters have both *on*-reading and *kun*-reading. A kanji character was rated as accurately read when either the *on*-reading or *kun*-reading was accurately named. Besides usual error analysis, we counted the occurrence of preceding *on*-reading and *kun*-reading deletion. That is, when a patient read a kanji character with *on*-reading, he was asked to read the character with *kun*-reading. This is because a kanji character is mostly read with *kun*-reading when presented in isolation (described in Discussion). Thus, reading first with *on*-reading is unusual in many cases. In addition, forgetting *kun*-reading directly represents a loss of semantics, as described in Introduction.

Furthermore, to determine the effects of visual complexity (measured by the number of writing stroke sequences), concreteness (Kitao et al., 1977), familiarity (Kitao et al., 1977), frequency (Amano and Kondo, 2000), and imageability (Sakuma et al., 2008) in writing a kanji character, we divided the test characters into two groups (above or under a median) nearly equal in number: a more complex (more writing stroke sequences), concrete, familiar, frequent or imageable group and a less complex, concrete, familiar, frequent, or imageable group. We compared the number of correct responses between the two groups.

Another reading test of two-character kanji called the *on*-*kun* reading test, consisting of 100 *on*-*on* reading words [e.g., 水泳 (sui-ei), swimming], 100 *kun*-*kun* reading words [e.g., 草花 (kusa-bana), grasses and flowers], and 60 specific-reading words called Jukujikun [e.g., 七夕 (tana-bata), star festival], was performed (Supplementary Appendix 2). All of the kanji characters were selected from those learned in primary and junior high schools in Japan.

The frequency was evaluated with articles covered in a Japanese newspaper from 1985 to 1998 (Amano and Kondo, 2000). Because the raw frequency values ranged from 1 to over 80,000, they were transformed to common logarithmic values. The mean transformed values were mutually different: 3.47 for *on*-reading, 2.64 for *kun*-reading, and 2.98 for specific-reading. On the other hand, the mean written-word familiarity values assessed on a seven-rating scale (Amano and Kondo, 2000) were also mutually different: 5.94 for *on*-reading, 5.62 for *kun*-reading, and 5.78 for specific-reading.

To control frequency and familiarity across the three types of reading tests for detailed analysis, we selected 60 words with consecutive or every-other frequency values from 100 word *on*- and *kun*-reading tests so that the mean and SD might be close to those of the specific reading test (Supplementary Appendix 2). Thus, the mean (SD) frequency was 2.97 (0.69) for on-reading, 2.95 (0.75) for *kun*-reading, and 2.98 (0.74) for specific-reading, whereas the mean familiarity (SD) was 5.85 (0.49) for on-reading, 5.72 (0.50) for *kun*-reading, and 5.78 (0.49) for specific-reading. To determine how frequency and familiarity influenced the correct responses, we further divided test words into two groups (above or under a median) nearly equal in number: a more frequent, familiar group and a less frequent, familiar group. We compared rate of correct responses (% correct) between the two groups.

### Neuroimaging Study

MRI T2-weighted images were obtained from eight patients (MRI images of patient 7 were missing). We assessed temporal lobe atrophy focusing on the dilatation of the inferior horn of the lateral ventricles. Imaging data on single photon emission computed tomography with a ^99m^Tc-ethylcysteinate dimer (^99m^Tc-ECD-SPECT) were obtained from seven patients (Patients 1 to 5, 8, and 9). First we compared each patient’s images with a normal subject database of the same generation and sex by the *t*-test (uncorrected *p* < 0.001), and then the seven patients’ data compared with each of the generation and sex groups were averaged using analysis of covariance (ANCOVA) by subject (multi-subject; condition (patient vs. healthy controls) by subject interaction, uncorrected *p* < 0.001). Inter-laboratory data correction was made possible with easy Z-score Imaging System (eZIS) (Matsuda et al., 2004). This system incorporates programs for realignment, spatial normalization, and smoothing from Statistical Parametric Mapping (Friston et al., 1995) version 2 (SPM2), and statistical analysis was conducted with SPM2 implemented in MATLAB 6.5.1. In each analysis, the extent threshold was set to be equal to the expected voxels per cluster to avoid noise clusters.

## Results

Patient profiles and neuropsychological data are shown in Table 1. In the WAB test, fluency, repetition, comprehension, reading, and writing were relatively preserved, whereas naming and kanji writing were impaired to various extents. Patient 5 achieved the highest scores in the WAB test and the *on–kun* reading test. However, he named a hammer “something to remove a staple.” A phonemic cue did not help. Even when the examiner taught him the correct answer, he did not identify it. This symptom was characteristic of semantic dementia and met a core feature of diagnostic criteria for semantic dementia (Neary et al., 1998; Gorno-Tempini et al., 2011). Also, the lowest score for the specific reading in the *on–kun* reading test suggested loss of semantics attached to the whole-word. The patient showed right-predominant temporal lobe atrophy, being consistent with the finding that anomia was less severe in patients with right-predominant atrophy than those with left-predominant atrophy (Woollams and Patterson, 2018).

### Kanji and Kana Reading and Writing Test

**FIGURE 1:**
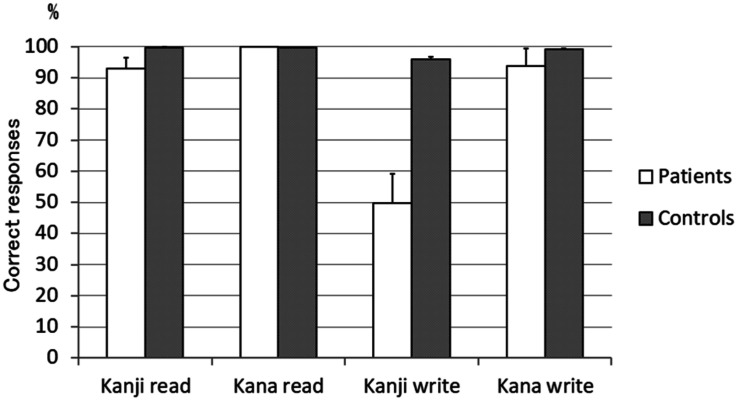
Mean rate of correct responses in the 100 single-character kanji and kana transcription test. Kanji writing was the most impaired. Kana writing and kanji reading were equally slightly impaired (*n* = 7). Error bar denotes standard error.

Mean correct responses in the kanji and kana reading and writing tests were the lowest for kanji writing, followed by kana writing and kanji reading (Figure 1). Errors of kanji reading were classified into visual (changing to a visually similar character), semantic (changing to a semantically related character), and phonological (phonemic paralexia), etc (Sakurai et al., 2000) according to a published English textbook (Coltheart, 1987). Error analysis revealed that in the kanji reading test, visual (18 out of a total of 33 errors) and semantic (8 out of 33 errors) errors accounted for most errors (details of the error types are shown in Supplementary Table S1). *On*-preceding was observed in all 7 patients (total of 92 characters), and *kun*-deletion was observed in 6 of the 7 patients (total of 32 characters). Errors of kanji writing were also classified into visual, semantic, and phonological, etc., similar to the reading (Supplementary Table S2). In the kanji writing test, no response was the most frequent (78% of all errors), and other orthographic errors including partial responses and visual errors were also frequent (10% of all errors).

Individual analysis of kanji writing revealed that the correct scores were significantly different in complexity (*p <* 0.01 by Fisher’s exact method) for 6 of 7 patients, frequency (*p* < 0.05 by Fisher’s exact method) for 4 of 7 patients, and familiarity (*p* < 0.01 by Fisher’s exact method) for 3 of 7 patients. That is, less complex, more frequent, and more familiar characters were written more easily, although these variables may not have been mutually independent: less complex kanji characters tended to be more frequent, familiar, and concrete.

### Kanji *On-Kun* Reading Test

**FIGURE 2:**
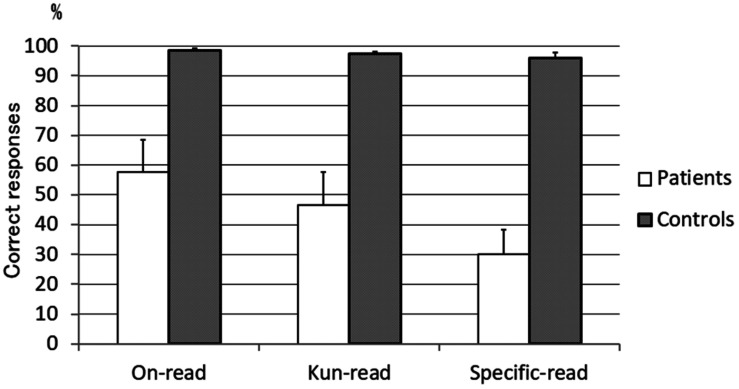
Mean rate of correct responses in the *on*-*kun* reading test. Specific-reading was the most impaired, then *kun*-reading, and *on*-reading was less impaired (*n* = 7). *On*-reading words = 100 words, *Kun*-reading words = 100 words, Specific-reading words = 60 words. Error bar denotes standard error.

The mean rate of correct responses for the *on*-*kun* reading test showed that *on*-reading (57.6%), *kun*-reading (46.6%), and specific-reading (30.0%) were more preserved in this decreasing order (Figure 2). A two-way repeated-measures analysis of variance (ANOVA) was conducted of the correct response rate, with reading type (*on*-*on* reading, *kun*-*kun* reading, and specific-reading) and group (patients vs. normal controls) as factors. The results showed the main effects of reading type [*F*(2,32) = 32.84, *p* < 0.001], group [*F*(1,16) = 46.59, *p* < 0.001], and interaction of reading type and group [*F*(2,32) = 22.73, *p* < 0.001]. A one-way repeated-measures ANOVA for reading type of the patient group also showed the main effect of reading type [*F*(2,12) = 19.52, *p* < 0.001], and post-hoc contrast revealed on-reading advantage over kun-reading (*p* = 0.034), *on*-reading advantage over specific-reading (*p* = 0.002) and *kun*-reading advantage over specific-reading (*p* = 0.006).

**FIGURE 3:**
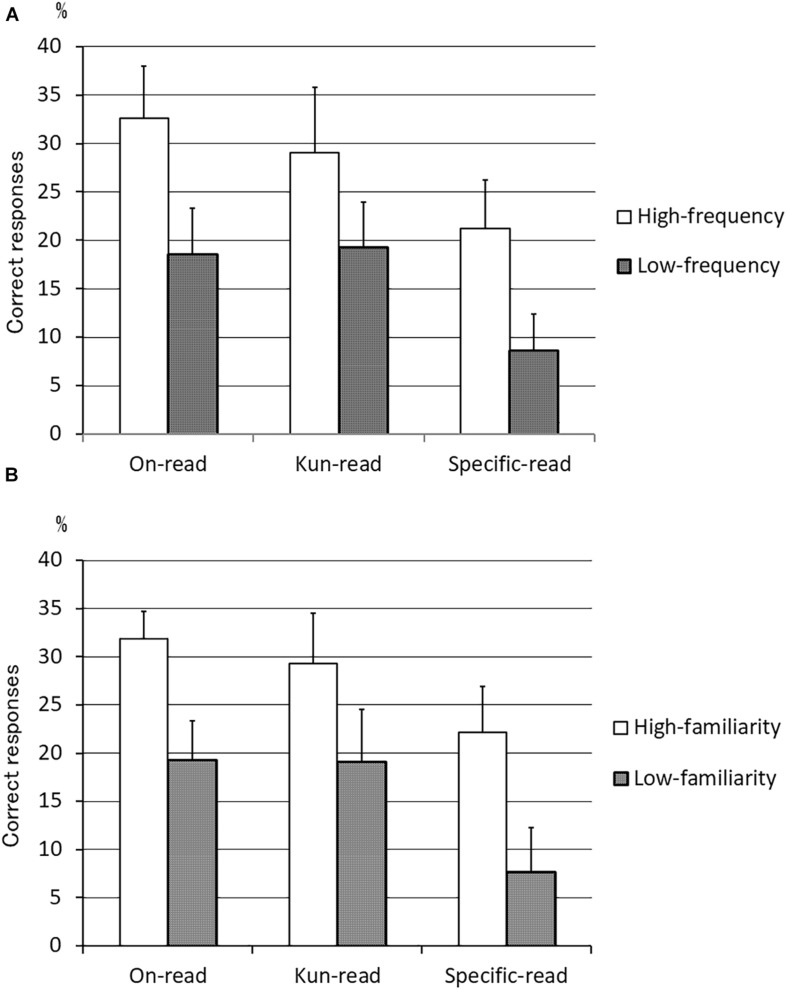
Mean rates of high- and low-frequency words and high- and low-familiarity words with correct responses in the *on-kun* reading test. **(A)** Mean % correct high- and low-frequency words. **(B)** Mean % correct high- and low-familiarity words. High-frequency or high-familiarity words were read more accurately than low-frequency or low-familiarity words in each reading test. *On*-reading predominance over *kun*-reading (not significant) and *kun*-reading predominance over specific-reading were observed only with high-frequency or high-familiarity words (*n* = 7). *On*-reading words = 60 words, *Kun*-reading words = 60 words, and Specific-reading words = 60 words. Error bar denotes standard error.

The mean % correct high- and low-frequency words and high- and low-familiarity words was calculated in the frequency- (and also familiarity-) matched 60 word *on*-reading, *kun*-reading, and specific-reading tests (Figure 3). As shown, high-frequency or high-familiarity words were read more accurately than low-frequency or low-familiarity words in each reading test. A two-way repeated-measures ANOVA for frequency and reading type showed the main effect of frequency [*F*(1,6) = 30.70, *p* = 0.001] and reading type [*F*(2,12) = 13.24, *p* = 0.001], without interaction. Post-hoc contrast revealed an *on*-reading advantage over specific-reading (*p* = 0.005) and a *kun*-reading advantage over specific-reading (*p* = 0.004), but not *on*-reading advantage over *kun*-reading. ANOVA for familiarity and reading type also showed the main effect of familiarity [*F*(1,6) = 48.27, *p* < 0.001] and reading type [*F*(2,12) = 13.59, *p* = 0.001], without interaction. Post-hoc contrast revealed an *on*-reading advantage over specific-reading (*p* = 0.005) and a *kun*-reading advantage over specific-reading (*p* = 0.004), but not *on*-reading advantage over *kun*-reading.

**FIGURE 4:**
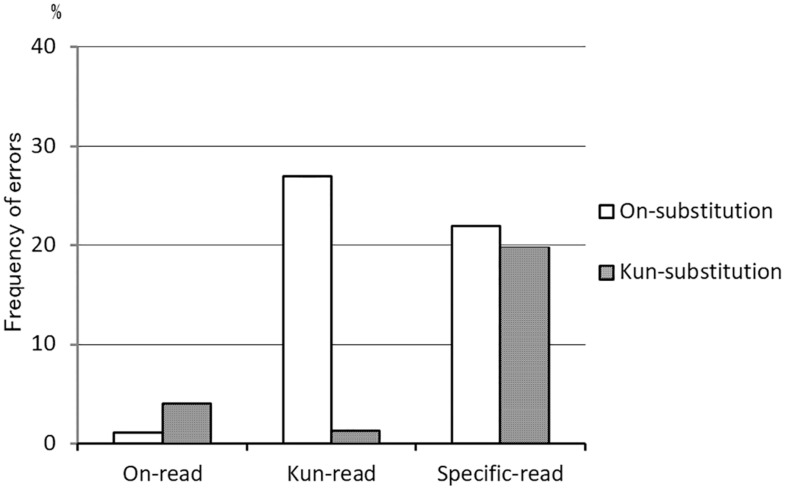
Frequency of substitution errors in the *on*-*kun* reading test. *On*-substitution errors in *kun*-reading words (27.0%) were more frequent than *kun*-substitution errors in *on*-reading words (4.0%) (*p* = 0.018 by Wilcoxon signed rank test). In specific-reading words, *on*- and *kun*-substitution errors were nearly equal in number (*n* = 7). *On*-reading words = 100 words, *Kun*-reading words = 100 words, and Specific-reading words = 60 words.

Next, we classified error types of reading, following those of the 100 kanji and kana reading and writing test (Sakurai, 2004) (Supplementary Table S3). Specifically, we examined the rate of substitution errors in each reading test (Figure 4). *On*-substitution denotes one or two substitutions of *on*-reading for *kun*-reading, e.g., 北風 [kita-kaze], *kun*-*kun* reading, meaning “north wind” → [hoku-huu], *on*-*on* reading, or 若者 [waka-mono], *kun*-*kun* reading, meaning “youth” → [waka-sha], *kun*-*on* reading. *Kun*-substitution denotes one or two substitutions of *kun*-reading for *on*-reading, e.g., 森林 [shin-rin], *on*-*on* reading, meaning “forest” → [mori-bayashi], *kun*-*kun* reading, or 親切 [shin-setsu], *on*-*on* reading, meaning “kindness” → [shin-kiri], *on*-*kun* reading. *On*-substitution in *kun*-*kun* reading words (27.0%) was significantly more frequent than *kun*-substitution in *on*-*on* reading words (4.0%) (*p* = 0.018 by Wilcoxon signed rank test). Also, *on*-substitution errors in *kun–kun* reading words were higher than that in specific-reading words, but not significant (*p* = 0.091 by Wilcoxon signed rank test). In specific-reading words, there was no significant difference of frequency between *on*-substitution errors and *kun*-substitution errors (*p* = 0.75 by Wilcoxon signed rank test).

### MRI and ECD-SPECT Findings

**FIGURE 5:**
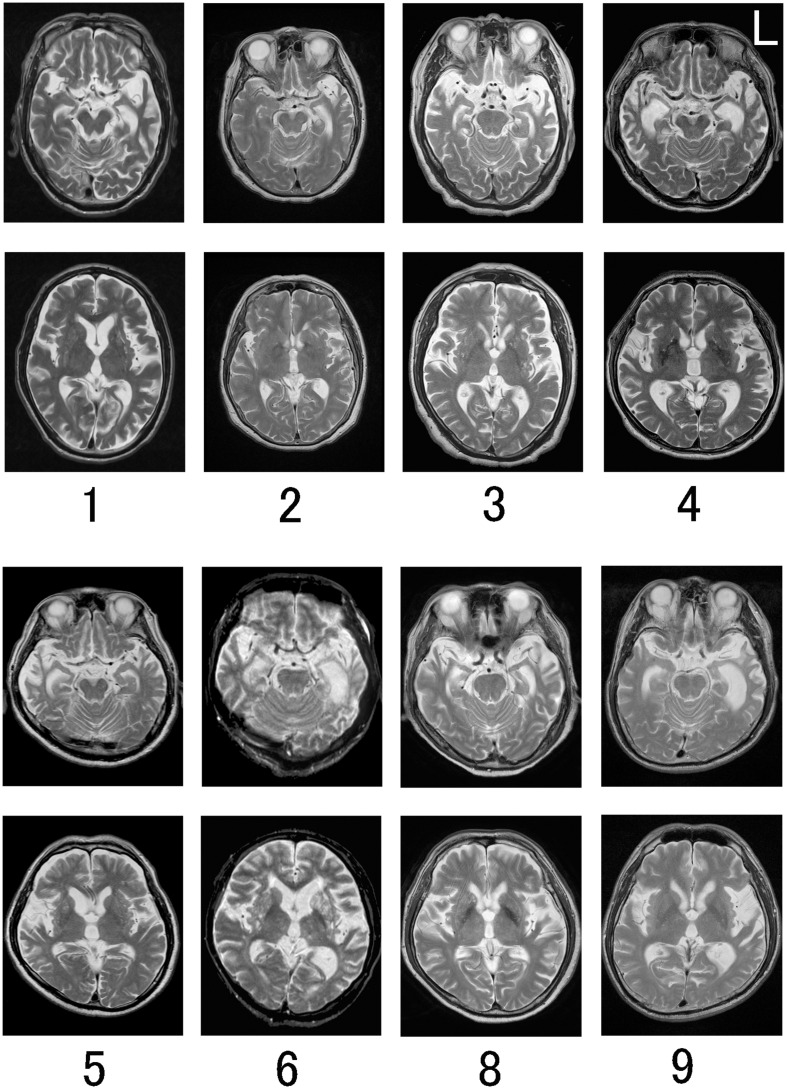
MRI T2-weighted images of eight semantic dementia patients. Number corresponds to the patient number in Table 1. MRI of patient 7 is not shown. Temporal lobe atrophy was left-sided only (Patients 1 and 2), left-right nearly equal (Patients 3, 4, and 8), right-predominant (Patient 5), and left-predominant (Patients 6 and 9).

**FIGURE 6:**
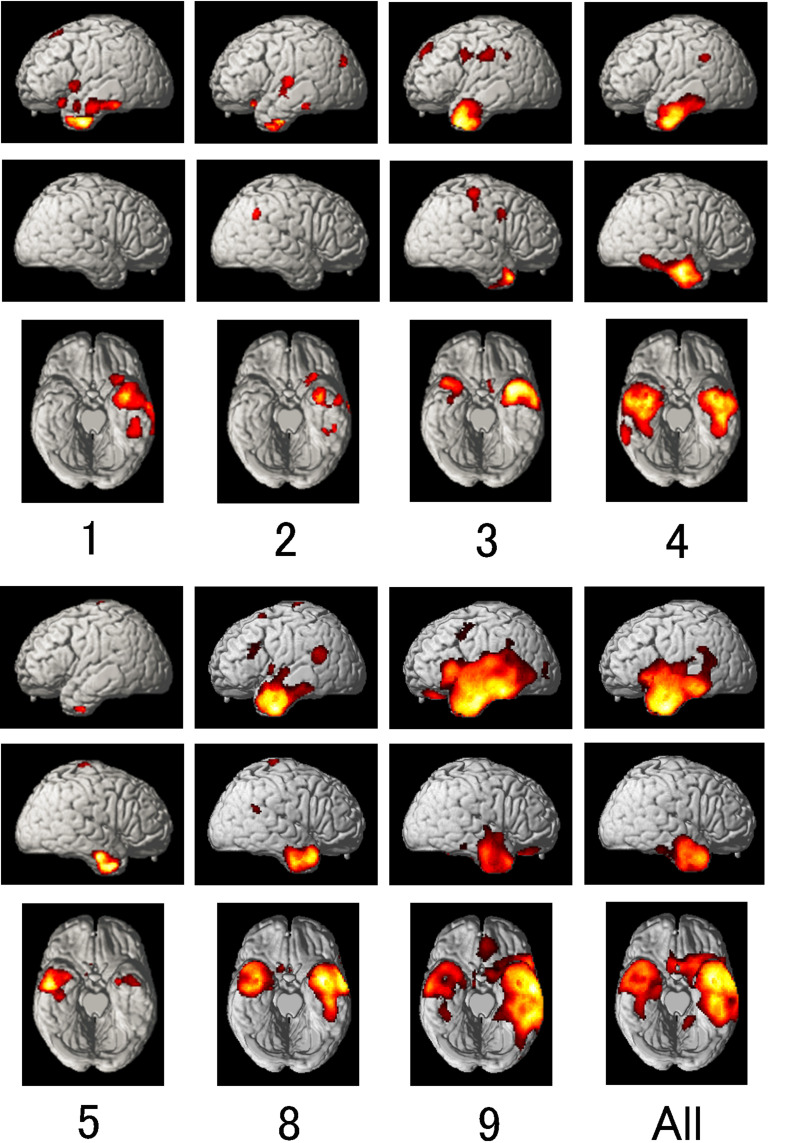
ECD-SPECT images of the 7 patients individually and altogether. Number corresponds to the patient number in Table 1. Each patient’s images were compared with those of normal subjects of the same generation and sex, using easy Z-score Imaging System (eZIS) and Statistical Parametric Mapping version 2 (SPM2). Areas of hypoperfusion (uncorrected *p* < 0.001 on *t*-test for individual analyses and analysis of covariance for all patients) are presented with a red-to-yellow gradient representing increasing z-scores. Temporal lobe hypoperfusion was left-predominant (Patients 1–3, 8, and 9), right-predominant (Patient 5), and left-right nearly equal (Patient 4). Averaged surface images revealed left-predominant hypoperfusion, involving the left posterior inferior frontal gyrus and anterior temporal lobe extending to the mid-fusiform gyrus. Areas with local maximum blood flow reduction in the temporal lobe were the left anterior and middle inferior temporal gyrus and mid-fusiform gyrus [Brodmann area (BA) 20; (–40, –4, –40), (–42, –24, –28), (–46, –42, –26)] and the right anterior temporal pole and middle and inferior temporal gyrus [BA 21; (42, 12, –32), (56, –8, –22), (42, –8, –34)] on the Montreal Neurological Institute (MNI) coordinate.

On MRI T2-weighted images, temporal lobe atrophy was left-sided only in three (patients 1, 2, and 7), left-right involvement was nearly equal in three (patients 3, 4, and 8), it was right-predominant in one (patient 5), and left-predominant in two (patients 6 and 9) (Table 1 and Figure 5). ECD-SPECT images showed temporal hypoperfusion that was nearly equivalent to the atrophy on MRI (Figure 6). Only two patients (3 and 8) showed left-predominant hypoperfusion in contrast to symmetrical temporal lobe atrophy. Averaged surface images revealed left-predominant hypoperfusion, involving the frontal operculum, and anterior temporal lobe extending to the mid-fusiform gyrus, suggesting that the lesion extension to the mid-fusiform gyrus is critical in producing anomia and alexia with agraphia in semantic dementia.

## Discussion

In the present study, *on*-preceding was observed in all 7 patients tested, and *kun*-deletion was observed in 6 of the 7 patients. This is consistent with our previous report that *on*-preceding and *kun*-deletion were characteristic of Japanese semantic dementia (Sakurai et al., 2006). In addition, *on*-reading, *kun*-reading, and specific-reading were significantly more preserved in this decreasing order (Figure 2). A more rigorous analysis after adjusting for word frequency (and familiarity) revealed that this trend, a kind of semantic effect that we call the phonology-to-semantics gradient, was not significant between *on*-reading and *kun*-reading. It is impossible, however, for the frequency or familiarity to directly create this gradient because the score for the *kun*-reading, whose mean frequency or familiarity was the lowest (described in Neuropsychological assessment), did not remain the lowest. Instead, it is possible that a reduction of test items from 100 to 60 is insufficient to detect the *on-kun* difference.

It is also possible that some patients do not exhibit *on*-reading superiority. Namely, the semantic dementia patients may comprise two groups: those in whom *kun*-reading is more impaired than *on*-reading (patients 2, 3, 4, and 8), and the others in whom *kun*-reading impairment is not so marked (patients 1, 5, and 9). We previously reported a patient with left temporal lobe hemorrhage who exhibited selective impairment of *on*-reading with preserved *kun*- and specific (Jukujikun) reading (Yoshida et al., 2020). This case, together with the present case of predominantly impaired *kun*-reading, supports a double dissociation between *on*-reading and *kun*-reading, and suggests that the phonological lexicon of *on*-reading and *kun*-reading is differently located in the temporal lobe. Taken together, it is possible that some semantic dementia patients exhibit *kun*-reading predominant impairment, whereas others exhibit not only *kun*-reading impairment but also *on*-reading impairment.

Although it is difficult to elucidate the relationship between the *on–kun* differences in reading and the temporal lobe atrophy/hypoperfusion, it is likely that *on*-reading predominance is observed in patients with left-only (patient 2), left-predominant (patients 3 and 8), or left-right nearly equal hypoperfusion (patient 4), whereas *on*-reading predominance is not so pronounced in patients with extensive temporal lobe hypoperfusion (patient 9), or right-predominant hypoperfusion (patient 5). Patient 1 with left-only hypoperfusion did not exhibit *on*-reading predominance, probably because the patient’s learning level of kanji may have confounded the test results.

Of particular note, *on*-substitution (changing to *on*-reading) errors in *kun*-reading words were significantly more frequent than *kun*-substitution (changing to *kun*-reading) errors in *on*-reading words (Figure 4). This result could be anticipated because two-character kanji words are mostly read with *on*-reading. For example, a standard Japanese kanji dictionary (Kimura and Kurosawa, 1996) contains 95.8% *on-on* reading, 3.1% *kun-kun* reading (including Jukujikun), 0.5% *on-kun* reading, and 0.6% *kun-on* reading words in a total of approximately 24,200 two-character kanji words excluding personal names. The *on*-substitution predominance (*on*-reading substitution errors are more frequent than *kun*-reading substitution errors) was also observed in the healthy subject group (Supplementary Table S3). However, the difference is that patients made more than six times as many *on*-substitution errors as healthy subjects (*on*/*kun* substitution ratio: 7 patients 189/9 vs. 11 controls 13/4) in the *kun*-reading word test. This is because patients did not recall the correct *kun*-reading, and thus depended on the residual *on*-reading. These findings suggest that *kun*-reading and specific-reading (Jukujikun) are more predominantly disturbed than *on*-reading in Japanese semantic dementia, probably because *kun*-reading is closely associated with the meaning of words, as described in Introduction. The same may apply to Western semantic dementia. Namely, patients with semantic dementia have difficulty reading irregular or exceptional words (surface dyslexia) because the pronunciation of these words inevitably involves accessing the original meaning, which patients progressively lose.

In summary, our study revealed that the *on*-*kun* difference had a distinct effect on the reading score. That is, the highest score for *on*-reading words with few *kun*-substitution errors suggests that *on*-reading, which primarily conveys phonetic values, was relatively preserved, whereas the relatively lower score for the *kun*-reading words with abundant *on*-substitution errors suggests that *kun*-reading, which directly links to the meaning, was selectively lost and the defect was compensated for with preserved *on*-reading. Moreover, the lowest score for specific-reading words suggests that specific-reading, of which the whole-word image directly links to the meaning, was markedly deteriorated and the defect was compensated for with either residual *on*- or *kun*-reading of the constituent kanji character.

The reason why there were not more *on*-substitution errors in the specific-reading test may be as follows. Although patients could not retrieve the whole-word reading, they obtained some semantic information from the combination of two characters. This vague semantic context effect (described in Introduction) may have helped to recall semantics-associated *kun*-reading of the individual kanji.

The *on*-*kun* confusion in Japanese semantic dementia suggests that the extent to which semantics is concerned with the pronunciation of the word (phonology-to-semantics gradient) determines the severity of surface dyslexia in semantic dementia.

### Relationship Between *On*-*Kun* Difference and Consistency

It has been shown that word frequency, familiarity, lexicality, and regularity (or consistency) influence the reading performance in semantic dementia (Jefferies et al., 2004; Patterson et al., 2006; Fushimi et al., 2009; Wilson et al., 2009; Playfoot et al., 2018). Characteristic is a frequency-by-regularity interaction in which the patients’ worst performance is on low-frequency irregular or exceptional words (Jefferies et al., 2004; Wilson et al., 2009). Similarly, in the Japanese language, a frequency-by-consistency interaction was reported (Fushimi et al., 2009). This phenomenon is explained as follows. Many kanji characters have multiple *on*-reading or *kun*-reading possibilities (Tamaoka et al., 2017). Fushimi et al. (2009) classified two-character kanji words into consistent (definite pronunciation in each position of a word), inconsistent typical (probable pronunciation), and inconsistent atypical (possible or improbable pronunciation), according to the probability of reading (frequency of being read in a Japanese dictionary), and reported that the frequency effect was more pronounced in this order of irregularity.

In our study, frequency also influenced the reading performance of *on*-reading words, *kun*-reading words, and specific-reading words (Figure 3). However, the reading type did not interact with frequency, which was attributed to the fact that there was little difference in the score between *on*-reading and *kun*-reading. This is partly because some semantic dementia patients did not exhibit *on*-reading predominance, as described in the previous session. Another factor associated with the lack of interaction is that the word lists were originally high-frequency words: we divided the words as relatively high frequency or relatively low frequency according to the median. Thus, the low-frequency words were actually “relatively lower” frequency words in a high-frequency word group. In order to precisely examine the interaction between reading type and frequency, we have to select high-frequency words and low-frequency words in a literal sense.

According to Fushimi et al.’s classification, our *on*-reading words are mostly inconsistent typical, and *kun*-reading and specific-reading words are mostly inconsistent atypical (Table 2A). In this regard, consistent words are, in fact, rare. Our study suggests that: (i) the distinction of inconsistent typical words and inconsistent atypical words or *on*-reading words and *kun*-reading words is not so robust: the difference was observed only in abundant 100 word lists; and (ii) inconsistent atypical words are further divided into *kun*-reading words and specific-reading (Jukujikun) words, on the basis of the fact that these two reading-types showed distinct reading performances in semantic dementia patients. This is because Jukujikun has an irregular *kun*-reading that cannot be predicted from the *kun*-reading of the two component characters (described in Introduction), and thus requires a direct link between the whole-word orthography and meaning. Although it is difficult to find words corresponding to Jukujikun in English, loanwords such as “yacht” may be similar to Jukujikun in that the pronunciation is extremely exceptional and requires a direct link between orthography and semantics.

**TABLE 2:** Numbers of reading-type responses.

	Consistent	Inconsistent t	Inconsistent at
**(A) This study**			
*On*-reading words (100)	25	72	3
*On*-reading words (60)	15	42	3
Hf words	8	23	0
Lf words	7	19	3
*Kun*-reading words (100)	0	5	95
*Kun*-reading words (60)	0	3	57
Hf words	0	2	28
Lf words	0	1	29
Specific-reading words (60)	0	0	60
Hf words	0	0	30
Lf words	0	0	30

	***On*-reading**	***Kun*-reading**	**Specific-reading**

**(B) Fushimi et al. (1999)**			
Hf consistent words (20)	20	0	0
Lf consistent word (20)	20	0	0
Hf inconsistent typical words (20)	20	0	0
Lf inconsistent typical words (20)	19	1	0
Hf inconsistent atypical words (20)	12^a^	7^a^	0
Lf inconsistent atypical words (20)	9	10	1

*^*a*^One word was an *on*-*kun* reading word, and was excluded from the list. Inconsistent t, inconsistent typical; Inconsistent at, inconsistent atypical; Hf, high-frequency; Lf, low-frequency.*

Conversely, in Fushimi et al.’s (1999) stimulus word list, consistent and inconsistent typical words comprised nearly all *on*-reading words, and inconsistent atypical words comprised *kun*-reading words more in low-frequency words (Table 2B). This fact suggests that the involvement of *kun*-reading and specific-reading words in the inconsistent atypical words contributed to reducing the reading performance.

It remains to be elucidated which is more important, consistency-inconsistency or *on-kun* difference, in reading words with semantic dementia. Roughly speaking, our *on*-reading words correspond to inconsistent typical words whereas *kun*-reading words correspond to inconsistent atypical words. However, this relationship is reversed when the Japanese read a single kanji character. That is, when kanji appear in compounds, they are expected to be pronounced with *on*-reading. In contrast, when kanji appear in isolation, they usually take a *kun*-reading (Kess and Miyamoto, 1999). Thus, when reading single characters, *kun*-reading becomes inconsistent typical, whereas *on*-reading becomes inconsistent atypical. The fact that patients show *on*-reading preceding and *kun*-reading deletion when reading single-character kanji implies that they read a kanji character with *on*-reading or inconsistent atypical (possible or improbable) reading. This is contradictory given that inconsistent typical reading is more frequently used than inconsistent atypical reading. It should be noted that consistent-inconsistent classification is applicable only to two-character kanji words whereas *on-kun* difference or phonology-to-semantics gradient is observed not only in two-character words but also in single-character words.

A limitation of the study is that there was no significant advantage for *on*-reading over *kun*-reading words, or no frequency-by-reading type interaction between *on*-reading and *kun*-reading, when the word frequency was matched. We have discussed the reason for these discrepant results, arguing that some semantic dementia patients exhibit predominant impairment of *kun*-reading whereas others exhibit *on*-reading impairment as well as *kun*-reading impairment, both of which may have different anatomical substrates. Further studies are required to determine that these two types of semantic dementia actually exist.

## Conclusion

*On*-reading (Chinese-style pronunciation) was relatively preserved whereas *kun*-reading (native Japanese pronunciation), particularly specific (Jukujikun) reading, was markedly disturbed in some Japanese semantic dementia patients. This fact, together with our previous case of selective impairment of *on*-reading (Yoshida et al., 2020), supports a double dissociation between the direct *on*-reading pathway from orthography (orthographic lexicon) to phonology (phonological lexicon) and the indirect *kun*-reading pathway from orthography to semantics, and then semantics to phonology.

This finding also suggests that Western semantic dementia patients have difficulty reading irregular or exceptional words primarily because these words require direct access to semantics, which patients progressively lose.

## Data Availability Statement

The original contributions presented in the study are included in the article/Supplementary Material, further inquiries can be directed to the corresponding author.

## Ethics Statement

The studies involving human participants were reviewed and approved by the Research Ethics Committee of the Mitsui Memorial Hospital. Written informed consent for participation was not required for this study in accordance with the institutional requirements. We used the official website of the Mitsui Memorial Hospital as an opt-out method.

## Author Contributions

YS, YU, AT, and YT made the diagnosis and collected data on each patient at their affiliations. YS prepared the manuscript. All authors agreed to be accountable for the content of the work.

## Conflict of Interest

The authors declare that the research was conducted in the absence of any commercial or financial relationships that could be construed as a potential conflict of interest.

## Publisher’s Note

All claims expressed in this article are solely those of the authors and do not necessarily represent those of their affiliated organizations, or those of the publisher, the editors and the reviewers. Any product that may be evaluated in this article, or claim that may be made by its manufacturer, is not guaranteed or endorsed by the publisher.
