# Supplementary material for: On-Reading (Chinese-Style Pronunciation) Predominance Over Kun-Reading (Native Japanese Pronunciation) in Japanese Semantic Dementia
Source: Front Hum Neurosci. 2021 Aug 5;15:700181. doi: 10.3389/fnhum.2021.700181 (PMC8374332; doi:10.3389/fnhum.2021.700181)
Supplement: Supplementary file 1 [file Data_Sheet_1.docx]

Supplementary Material

Appendix 1. Single-character kanji and kana transcription reading and writing test.

Kanji characters

山　月　犬　花　雨　空　車　星　力　下　音　右　年　男　左

入　立　生　休　見　学　出　知　小　大　正　早　赤　青　白

牛　竹　米　海　家　雲　道　顔　今　冬　西　夏　数　話　昼

行　会　来　買　読　聞　作　飲　太　広　古　弱　強　黒　長

坂　水　岩　岸　鳥　庭　駅　港　列　角　味　旅　緑　横　色

打　死　言　勝　運　遊　書　動　苦　重　悲　悪　軽　寒　多

指　前　朝　外　親　消　高　近　深　細

Kana transcription (kana sequences of a word were assigned vertically)

や つ い は あ そ く ほ ち し お み と お ひ

ま き ぬ な め ら る し か た と ぎ し と だ

ま ら こ り

は た い や み ま で し ち お た は あ あ し

い つ き す る な る る い お だ や か お ろ

る る む ぶ さ き し い い い い

い い い

う た こ う い く み か い ふ に な か は ひ

し け め み え も ち お ま ゆ し つ ず な る

し

い あ く か よ き つ の ふ ひ ふ よ つ く な

く う る う む く く む と ろ る わ よ ろ が

る い い い い い い い

さ み い き と に え み れ か あ た み よ い

か ず わ し り わ き な つ ど じ び ど こ ろ

と り

う し い か は あ か う く お か わ か さ お

つ ぬ う つ こ そ く ご る も な る る む お

ぶ ぶ く し い し い い い い

い い

ゆ ま あ そ お け た ち ふ ほ

び え さ と や す か か か そ

い い い い
