# Supplementary material for: On-Reading (Chinese-Style Pronunciation) Predominance Over Kun-Reading (Native Japanese Pronunciation) in Japanese Semantic Dementia
Source: Front Hum Neurosci. 2021 Aug 5;15:700181. doi: 10.3389/fnhum.2021.700181 (PMC8374332; doi:10.3389/fnhum.2021.700181)
Supplement: Supplementary file 2 [file Data_Sheet_2.docx]

Supplementary Material

Appendix 2. Two-character *on*-*kun* reading test.

*On*-*on*-reading words (100 words)

水泳　　　　 海岸　　　　 朝食　　　　 森林　　　　 石油

車輪　　　　 牛乳　　　　 明暗　　　　 強弱　　　　 同時

近所　　　　 人数　　　　 外国　　　　 見学　　　　 目次

正月　　　　 有名　　　　 勝負　　　　 集合　　　　 返事

前後　　　　 中止　　　　 動物　　　　 調子　　　　 旅行

心配　　　　 開始　　　　 目的　　　　 印象　　　　 急速

変化　　　　 習慣　　　　 責任　　　　 指導　　　　 姿勢

補助　　　　 貧富　　　　 歩道　　　　 会話　　　　 親切

野菜　　　　 神経　　　　 競技　　　　 尊敬　　　　 貴重

危険　　　　 難問　　　　 妻子　　　　 確認　　　　 誠実

空腹　　　　 善悪　　　　 負傷　　　　 頭痛　　　　 分割

閉店　　　　 盛大　　　　 著者　　　　 方針　　　　 胸囲

降参　　　　 解除　　　　 主従　　　 価値　　　 伝染

断水　　　　 沿道　　　　 巻末　　　　 担当　　　　 拝見

延長　　　　 国宝　　　　 時刻　　　　 呼吸　　　　 近似

健忘　　　　 産卵　　　　 困難　　　　 乱筆　　　　 増減

石灰　　　　 破片　　　　 亡命　　　　 組織　　　　 燃焼

建築　　　　 正確　　　　 仏教　　　　 綿花　　　　 飼育

預金　　　　 豊富　　　　 犯罪　　　　 幹事　　　　 勢力

過程　　　　 絶望　　　　 測定　　　　 減少　　　　 移動

Selected *on*-*on*-reading words (60 words)

水泳　　　　 海岸　　　　 朝食　　　　 森林　　　　 車輪

牛乳　　　　 明暗　　　　 強弱　　　　 同時　　　　 人数

見学　　　　 目次　　　　 正月　　　　 有名　　　　 集合

返事　　　　 急速　　　　 習慣　　　　 貧富　　　　 歩道

会話　　　　 神経　　　　 尊敬　　　　 貴重　　　　 難問

妻子　　　　 誠実　　　　 空腹　　　　 善悪　　　　 頭痛

閉店　　　　 盛大　　　　 胸囲　　　　 降参　　　　 主従

伝染　　　　 断水　　　　 沿道　　　　 巻末　　　　 拝見

国宝　　　　 時刻　　　　 呼吸　　　　 近似　　　　 健忘

産卵　　　　 乱筆　　　　 増減　　　　 石灰　　　　 破片

亡命　　　　 燃焼　　　　 正確　　　　 仏教　　　　 綿花

飼育　　　　 豊富　　　　 幹事　　　　 絶望　　　　 測定

*Kun*-*kun*-reading words (100 words)

草花　　　　 北風　　　　 広場　　　　 雨雲　　　　 坂道

野原　　　　 谷底　　　　 海辺　　　　 米俵　　　　 口紅

灰色　　　　 小麦　　　　 上着　　　　 若者　　　　 宿屋

目印　　　　 物音　　　　 建物　　　　 昼間　　　　 親子

朝市　　　　 割引　　　　 骨身　　　　 針金　　　　 品切

小銭　　　　 書留　　　　 昔話　　　　 厚手　　　　 積立

塩焼　　　　 古巣　　　　 裏側　　　　 島国　　　　 遠浅

型紙　　　　 花束　　　　 指輪　　　　 名札　　　　 小包

氏神　　　　 石橋　　　　 鼻歌　　　　 横顔　　　　 虫歯

港町　　　　 植木　　　　 真心　　　　 旅人　　　　 中庭

重荷　　　　 角笛　　　　 相手　　　　 神主　　　　 炭火

屋根　　　　 品物　　　　 悪者　　　　 波間　　　　 父親

川岸　　　　 取組　　　　 黒豆　　　　 羽衣　　　　 名前

子守　　　　 安値　　　　 毛皮　　　　 小皿　　　　 申込

打切　　　　 氷水　　　　 顔色　　　　 物語　　　　 歌声

居間　　　　 花園　　　　 買手　　　　 朝日　　　　 場合

近道　　　　 晴着　　　　 小鳥　　　　 黄緑　　　　 黒山

雪国　　　　 魚屋　　　　 組合　　　　 夕立　　　　 手紙

青空　　　　 大通　　　　 弱虫　　　　 生水　　　　 風上

砂浜　　　　 門出　　　　 夜店　　　　 月夜　　　　 人里

Selected *kun*-*kun*-reading words (60 words)

草花　　　　 広場　　　　 野原　　　　 谷底　　　　 海辺

米俵　　　　 口紅　　　　 灰色　　　　 小麦　　　　 上着

若者　　　　 物音　　　　 建物　　　　 昼間　　　　 親子

割引　　　　 骨身　　　　 針金　　　　 書留　　　　 昔話

積立　　　　 裏側　　　　 遠浅　　　　 花束　　　　 指輪

小包　　　　 鼻歌　　　　 横顔　　　　 港町　　　　 植木

重荷　　　　 相手　　　　 屋根　　　　 品物　　　　 悪者

波間　　　　 父親　　　　 黒豆　　　　 名前　　　　 子守

安値　　　　 毛皮　　　　 小皿　　　　 申込　　　　 打切

物語　　　　 歌声　　　　 居間　　　　 花園　　　　 朝日

小鳥　　　　 黄緑　　　　 組合　　　　 夕立　　　　 手紙

青空　　　　 大通　　　　 門出　　　　 夜店　　　　 人里

Specific-reading (Jukujikun) words (60 words)

七夕　　　　 上手　　　　 眼鏡　　　　 従兄　　　　 大人

迷子　　　　 素人　　　　 足袋　　　　 土産　　　　 田舎

小豆　　　　 景色　　　　 部屋　　　　 相撲　　　　 納得

下手　　　　 布団　　　　 果物　　　　 大豆　　　　 梅雨

大和　　　　 明日　　　　 支度　　　　 昨日　　　　 夏至

笑顔　　　　 乙女　　　　 心地　　　　 今年　　　　 砂利

師走　　　　 吹雪　　　　 息子　　　　 真青　　　　 行方

浮気　　　　 伯父　　　　 風邪　　　　 仮名　　　　 河原

玄人　　　　 芝生　　　　 今日　　　　 友達　　　　 二人

一日　　　　 木綿　　　　 叔母　　　　 二日　　　　 紅葉

時雨　　　　 竹刀　　　　 真赤　　　　 硫黄　　　　 乳母

母屋　　　　 為替　　　　 今朝　　　　 数珠　　　　 雪崩
