# Supplementary material for: On-Reading (Chinese-Style Pronunciation) Predominance Over Kun-Reading (Native Japanese Pronunciation) in Japanese Semantic Dementia
Source: Front Hum Neurosci. 2021 Aug 5;15:700181. doi: 10.3389/fnhum.2021.700181 (PMC8374332; doi:10.3389/fnhum.2021.700181)
Supplement: Supplementary file 3 [file Table_1.docx]

Supplementary Material

Table S1. Types of errors in 100 single-character kanji reading.

Patient 1 2 3 4 5 6 7 HC^a^

----------------------------------------------------------------------------------------------------------

No response 0 0 0 0 0 1 0 0

Lexical 0 0 0 0 0 0 0 1

Semantic 0 4 1 0 0 1 2 1

Visual 0 4 5 2 0 3 4 0

Phonological 0 1 0 0 0 0 5 1

Unrelated 0 0 0 0 0 0 0 1

----------------------------------------------------------------------------------------------------------

Total 0 9 6 2 0 5 11 4

*On*-preceding 11 8 26 1 9 27 10 2

*Kun*-deletion 4 2 ?^b^ 5 5 9 7 0

----------------------------------------------------------------------------------------------------------

Visual: changing the kanji reading to the reading of another kanji with a visual resemblance to the correct answer, e.g., 読 ([yomu], read) → read as 続 ([tsuduku], continue), Lexical: changing to the reading of another word that contains the stimulus character, e.g., 味 ([mi], taste) → read as 意味 ([imi], meaning), Semantic: changing the reading to another reading associated semantically with the target word, e.g., 列 ([retsu], line) → read as 長 ([nagai], long), Phonological: one or more phonemes of a kanji word were substituted for other phonemes (phonemic paralexia), e.g., 来 ([kuru], come) → [saru], Unrelated: changing to the reading of another word that has no phonological or orthographical similarity, *On*-preceding: patients read a kanji character first with *on*-reading, irrespective of the context. *Kun*-deletion: pronouncing a kanji character with on-reading is possible, but not with *kun*-reading.

^a^Total number of errors for 11 healthy controls (HC). They were ten men and one woman, aged 61–78 (mean: 68 years), with 12 or more year’s education and no past history of neurological disorders.

^b^The examiner did not ask about how the word that was first read by *on*-reading could also be pronounced by *kun*-reading. Thus, *kun*-reading deletion was not ascertained.
