# Supplementary material for: On-Reading (Chinese-Style Pronunciation) Predominance Over Kun-Reading (Native Japanese Pronunciation) in Japanese Semantic Dementia
Source: Front Hum Neurosci. 2021 Aug 5;15:700181. doi: 10.3389/fnhum.2021.700181 (PMC8374332; doi:10.3389/fnhum.2021.700181)
Supplement: Supplementary file 4 [file Table_2.docx]

Supplementary Material

Table S2. Types of errors in 100 single-character kanji writing.

Patient 1 2 3 4 5 6 7 HC^a^

----------------------------------------------------------------------------------------------------------

No response 84 35 34 14 10 43 55 26

Partial response 2 3 5 0 3 0 0 6

Constructional 3 0 0 0 1 1 0 5

Semantic 1 0 1 1 0 0 1 2

Visual 0 5 2 4 1 0 4 1

Phonological 0 3 0 4 0 0 5 0

Phonological/semantic 0 0 0 1 0 0 0 0

Neographism 0 3 1 0 0 1 0 2

Unrelated 0 11 1 5 0 1 3 1

----------------------------------------------------------------------------------------------------------

Total 90 60 44 29 15 46 68 43

----------------------------------------------------------------------------------------------------------

Partial response: a component of a kanji character is correct, e.g., 緑 ([midori], green) → 終 ([owari], end), Constructional: the omission or addition of a stroke of a kanji e.g., 車 ([kuruma], wheel) →

, Semantic: substitution of another semantically associated character, e.g., 月 ([tsuki], moon) → 日 ([hi], sun), Visual: substitution of another visually similar character. e.g., 西 ([nishi], west) → 面 ([men], face), Phonological: changing to another kanji with one phoneme substitution, e.g., 勝 ([katsu], win) → 各 ([kaku], each), Phonological/semantic: substitution of another kanji phonologically and semantically associated with the target character, e.g., 多 ([ooi], numerous) → 大 ([ookii], large), Unrelated: substitution of another kanji that has no visual or phonological similarity with the correct answer.

^a^Total number of errors for 11 healthy controls (HC). They were the same as those in Table S1. One raw datum (including two errors) was missing.
