# Supplementary material for: On-Reading (Chinese-Style Pronunciation) Predominance Over Kun-Reading (Native Japanese Pronunciation) in Japanese Semantic Dementia
Source: Front Hum Neurosci. 2021 Aug 5;15:700181. doi: 10.3389/fnhum.2021.700181 (PMC8374332; doi:10.3389/fnhum.2021.700181)
Supplement: Supplementary file 5 [file Table_3.docx]

Supplementary Material

Table S3. Types of errors in *on-kun* reading test

Patient 1 2 3 4 5 8 9 HC^a^

----------------------------------------------------------------------------------------------------------

*On*-reading

No response 7 0 4 1 0 32 16 2

Partial response 4 0 2 0 0 20 1 0

Phonological 4 27 10 4 4 9 28 2

*On*-substitution 1 1 1 1 1 1 1 0

*On*-substitution/visual 0 0 0 0 0 1 0 0

*Kun*-substitution 4 6 2 2 1 2 10 1

*Kun*-substitution/visual 0 0 0 0 0 0 1 0

Lexical 6 7 9 4 6 5 2 9

Semantic 1 1 4 0 0 2 1 1

Visual 2 1 0 1 0 1 0 0

Visual/phonological 0 2 0 0 0 0 0 0

Unrelated 0 9 0 0 0 7 17 0

*Kun*-reading

No response 8 0 4 1 0 22 3 4

Partial response 4 0 4 0 0 15 2 1

Phonological 1 18 3 7 3 1 20 1

*On*-substitution 14 46 40 22 6 27 32 13

*On-substitution/visual* 0 0 0 0 0 0 2 0

*Kun*-substitution 2 1 0 1 3 1 1 4

Lexical 0 4 2 3 0 5 0 6

Semantic 0 0 0 1 0 3 2 1

Visual 0 2 2 2 0 0 8 0

Unrelated 0 0 0 0 0 17 9 0

Specific-reading

No response 23 0 5 2 0 18 4 3

Partial response 0 0 2 0 0 4 0 0

Phonological 1 5 3 0 2 0 11 0

*On*-substitution 5 27 14 17 7 13 14 10

*Kun*-substitution 10 12 15 17 7 16 15 4

*Kun*-/*on*-substitution 0 0 0 1 0 0 1 0

Lexical 0 5 3 0 0 0 0 6

Semantic 0 0 0 0 0 3 2 1

Visual 0 0 0 0 1 0 0 0

Unrelated 0 0 0 0 0 3 7 0

----------------------------------------------------------------------------------------------------------

Partial response: only one character was read correctly, Phonological: phonemic paralexia, *On*-substitution: changing *on*-reading to another *on*-reading in the *on*-reading test, *kun*-reading to *on*-reading in the kun-reading test, and specific-reading to *on*-reading in the specific-reading test. *Kun*-substitution: changing *on*-reading to *kun*-reading in the *on*-reading test, *kun*-reading to another *kun*-reading in the *kun*-reading test, and specific-reading to *kun*-reading in the specific-reading test. Lexical: changing to another word that contains one kanji character of the correct word, e.g., 過程 ([katei], process) → read as 過度 ([kado], excess), Visual/phonological: one character is read as a visually similar character, and another character is pronounced incorrectly, *Kun*-/*on*-substitution: one character has *kun*-substitution and another character has *on*-substitution. For other errors, see the footnote of Table S1.

^a^Total number of errors for 11 healthy controls (HC). They were ten men and one woman, aged between 64 and 84 (mean: 74 years), with 9 or more years’ education and no past history of neurological disorders. They were not the same as the normal subjects for the ECD-SPECT study.
